# Copper activates HIF-1α/GPER/VEGF signalling in cancer cells

**DOI:** 10.18632/oncotarget.5779

**Published:** 2015-09-22

**Authors:** Damiano Cosimo Rigiracciolo, Andrea Scarpelli, Rosamaria Lappano, Assunta Pisano, Maria Francesca Santolla, Paola De Marco, Francesca Cirillo, Anna Rita Cappello, Vincenza Dolce, Antonino Belfiore, Marcello Maggiolini, Ernestina Marianna De Francesco

**Affiliations:** ^1^ Department of Pharmacy, Health and Nutritional Sciences, University of Calabria, Rende, Italy; ^2^ Endocrinology, Department of Health, University Magna Graecia of Catanzaro, Catanzaro, Italy

**Keywords:** copper, cancer, angiogenesis, GPER, HIF-1α, VEGF, Pathology Section

## Abstract

Copper promotes tumor angiogenesis, nevertheless the mechanisms involved remain to be fully understood. We have recently demonstrated that the G-protein estrogen receptor (GPER) cooperates with hypoxia inducible factor-1α (HIF-1α) toward the regulation of the pro-angiogenic factor VEGF. Here, we show that copper sulfate (CuSO_4_) induces the expression of HIF-1α as well as GPER and VEGF in breast and hepatic cancer cells through the activation of the EGFR/ERK/c-fos transduction pathway. Worthy, the copper chelating agent TEPA and the ROS scavenger NAC prevented the aforementioned stimulatory effects. We also ascertained that HIF-1α and GPER are required for the transcriptional activation of VEGF induced by CuSO_4_. In addition, in human endothelial cells, the conditioned medium from breast cancer cells treated with CuSO_4_ promoted cell migration and tube formation through HIF-1α and GPER.

The present results provide novel insights into the molecular mechanisms involved by copper in triggering angiogenesis and tumor progression. Our data broaden the therapeutic potential of copper chelating agents against tumor angiogenesis and progression.

## INTRODUCTION

Copper, which is an essential trace element naturally occurring in soil, water and air, acts as a catalytic and/or structural cofactor in a wide array of important biological processes like embryogenesis, growth, homeostasis and angiogenesis [1, 2]. An elevated exposure to copper may be mainly consequent to environmental pollution from the manufacture of wire, sheet metal, pipe and other metal products [2]. In addition, mining, waste dumps, combustion of fossil fuels, wood production and phosphate fertilizers release copper in the environment, thus contributing to the actual exposure in humans [2-4]. To date, mismanaged or high copper levels have been involved in the generation of oxidative stress [5] which plays an important role in cancer development [6]. In this regard, it should be mentioned that physiological concentrations of copper range approximately from 18 to 31 μM [7], while serum copper levels have been found in cancer patients from 50 μM to 205 μM or even at mM concentrations [8-10]. Of note, elevated copper concentrations were correlated with cancer stage and/or progression in diverse types of tumors, thus suggesting that copper may be a useful prognostic factor and a marker of responsiveness to therapy [reviewed in 8]. On the basis of these findings, a number of studies investigated the stimulatory action of copper on VEGF production and tumor angiogenesis [11-13] and the repressive effects exerted by copper-chelating on HIF-1α mediated expression of VEGF [14, 15].

Numerous G-protein coupled receptors (GPCRs) contribute to the angiogenic switch through mechanisms that include their functional interaction with HIF-1α toward VEGF expression [16]. In this regard, our recent study has shown that the G protein estrogen receptor (GPER) cooperates with HIF-1α in order to modulate VEGF in hypoxic breast tumor microenvironment [17]. In addition, we have demonstrated that estrogenic GPER signalling activates HIF-1α/VEGF transduction pathway leading to angiogenesis and tumor growth [18].

Here, we provide novel evidence on the mechanisms by which copper triggers the EGFR/ERK/c-fos signalling cascade along with GPER and HIF-1α toward VEGF expression and function in cancer cells. We also show that GPER may be considered as an additional target of copper chelating agents, hence broadening the therapeutic potential of these chemicals against tumor angiogenesis and progression.

## RESULTS

### CuSO_4_ induces the expression of the pro-angiogenic factor VEGF

**Figure 1:**
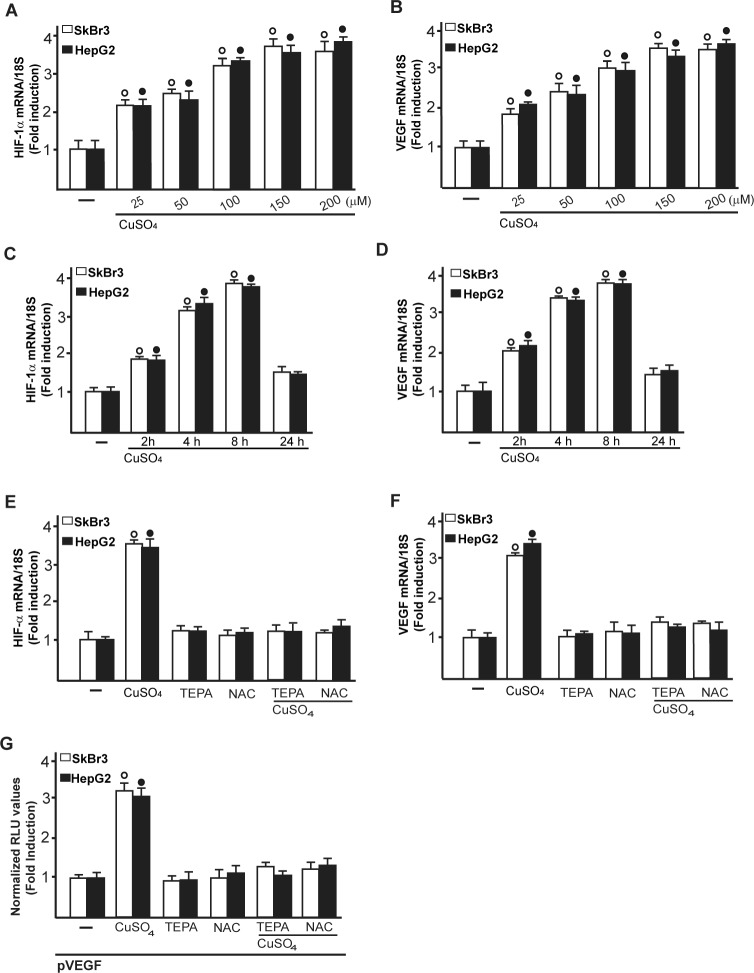
CuSO_4_ induces the mRNA expression of HIF-1α and VEGF mRNA expression of HIF-1α **A.** and VEGF **B.** in SkBr3 and HepG2 cells treated with increasing concentrations of CuSO_4_ for 8 hours, as evaluated by real-time PCR. CuSO_4_ (200 μM) induces the mRNA expression of HIF-1α **C.** and VEGF **D.** in a time-dependent manner. In SkBr3 and HepG2 cells treated with 200 μM CuSO_4_ for 8 hours, the mRNA induction of HIF-1α **E.** and VEGF **F.** is abrogated in the presence of the copper chelating agent TEPA (50 μM) and the ROS scavenger NAC (300 μM). Values are normalized to the 18S expression and shown as fold changes of the mRNA expression induced by CuSO_4_ compared to cells treated with vehicle (−). **G.** The transactivation of a VEGF promoter plasmid (pVEGF) observed in SkBr3 and HepG2 cells treated with 200 μM CuSO_4_ for 12 hours is prevented by TEPA (50 μM) and NAC (300 μM). The luciferase activities were normalized to the internal transfection control and values of cells receiving vehicle (−) were set as 1-fold induction upon which the activities induced by CuSO_4_ treatment were calculated. Each data point represents the mean ± SD of three independent experiments performed in triplicate. (○), (●) *p* < 0.05 for cells receiving vehicle (−) *versus* CuSO_4_ treatment.

**Figure 2:**
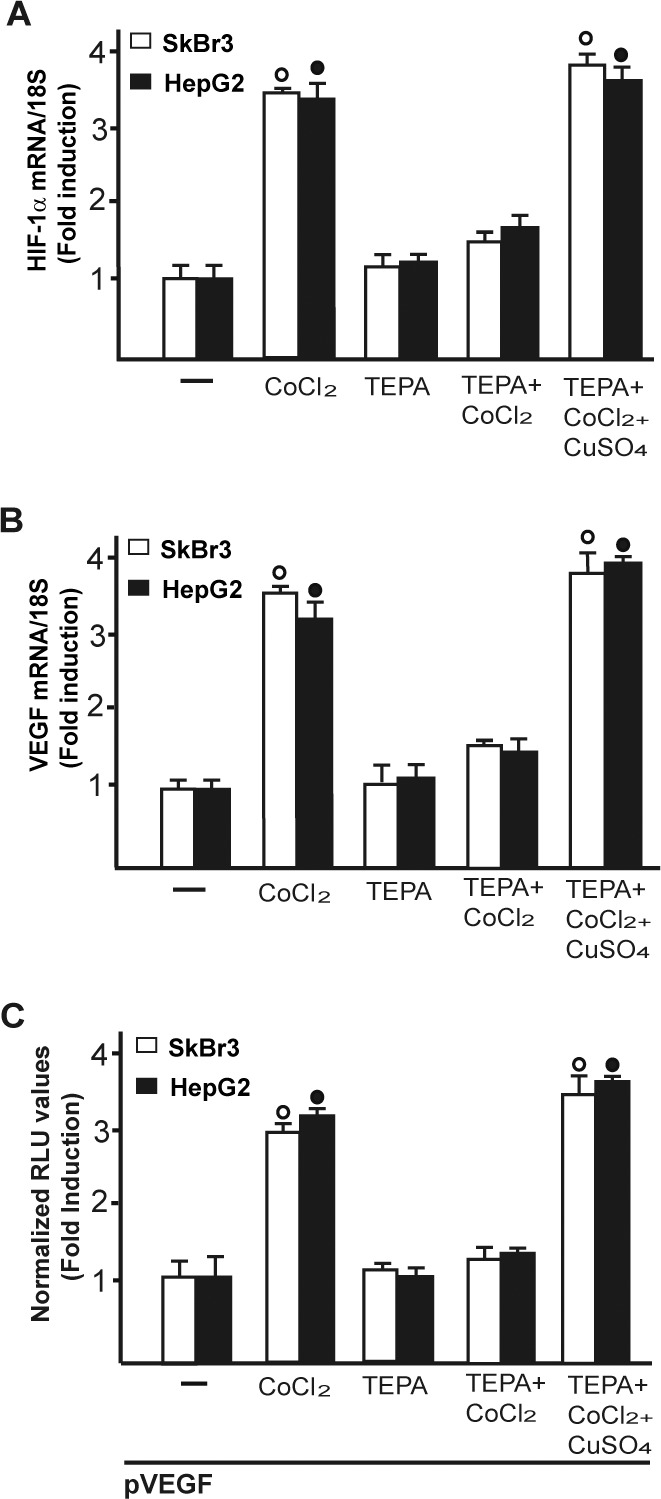
CuSO_4_ rescues the inhibitory effects of TEPA on CoCl_2_-induced transcription of HIF-1α and VEGF In SkBr3 and HepG2 cells, the up-regulation of HIF-1α **A.** and VEGF **B.** mRNA expression induced upon CoCl_2_ treatment (100 μM for 8 hours) is no longer evident in the presence of TEPA (50 μM) but rescued using CoCl_2_ (100 μM for 8 hours) in combination with 200 μM CuSO_4_, as determined by real-time PCR. Values are normalized to the 18S expression and shown as fold changes of mRNA expression induced by treatments respect to cells treated with vehicle (−). **C.** The transactivation of a VEGF promoter plasmid (pVEGF) observed in SkBr3 and HepG2 cells treated with 100 μM CoCl_2_ for 12 hours is prevented by TEPA (50 μM) and rescued using CoCl_2_ (100 μM for 12 hours) in combination with 200 μM CuSO_4_. The luciferase activities were normalized to the internal transfection control and values of cells receiving vehicle (−) were set as 1-fold induction upon which the activities induced by CoCl_2_ treatment were calculated. Each data point represents the mean ± SD of three independent experiments performed in triplicate. (○), (●) *p* < 0.05 for cells receiving vehicle (−) *versus* treatments.

Considering that copper and its chelating agents have been involved in tumor angiogenesis [5], we asked whether copper sulfate (CuSO_4_) may induce the expression of the pro-angiogenic factor VEGF and its transcriptional regulator HIF-1α in SkBr3 breast cancer cells and HepG2 hepatocellular carcinoma cells. Of note, CuSO_4_ induced the mRNA expression of both HIF-1α (Figure 1A) and VEGF (Figure 1B) in a dose dependent manner, starting from 25 μM and reaching the strongest stimulation upon concentrations ranging from 100 to 200 μM. Taking into account these results and considering that in previous studies relevant biological responses to copper exposure were observed up to 500 μM [19-21], in the subsequent assays of the current study 200 μM CuSO_4_ were used. First, we determined that CuSO_4_ up-regulates in a time-dependent manner the mRNA expression of HIF-1α (Figure 1C) and VEGF (Figure 1D) in SkBr3 and HepG2 cells. Thereafter, we ascertained that the well-acknowledged copper chelating agent TEPA [14, 22] as well as the extensively used ROS scavenger NAC [reviewed in 23] prevent the mRNA induction of HIF-1α (Figure 1E) and VEGF (Figure 1F) and the transactivation of a VEGF promoter construct (Figure 1G) upon treatment with CuSO_4_. As copper has been previously involved in HIF-1α responses to low oxygen conditions [14, 15], we then assessed the effect of TEPA on the action of the hypoxia-mimetic agent CoCl_2_. As expected, CoCl_2_ induced the mRNA expression of HIF-1α (Figure 2A) and VEGF (Figure 2B) as well as the transactivation of a VEGF promoter construct (Figure 2C) in SkBr3 and HepG2 cells. Interestingly, these effects were abolished in the presence of TEPA and rescued adding CuSO_4_ to SkBr3 and HepG2 cells (Figure 2). Results similar to those observed using CoCl_2_ were obtained culturing SkBr3 and HepG2 cells in a low oxygen tension (2% O_2_) (Supplementary Figure 1A-1C).

**Figure 3:**
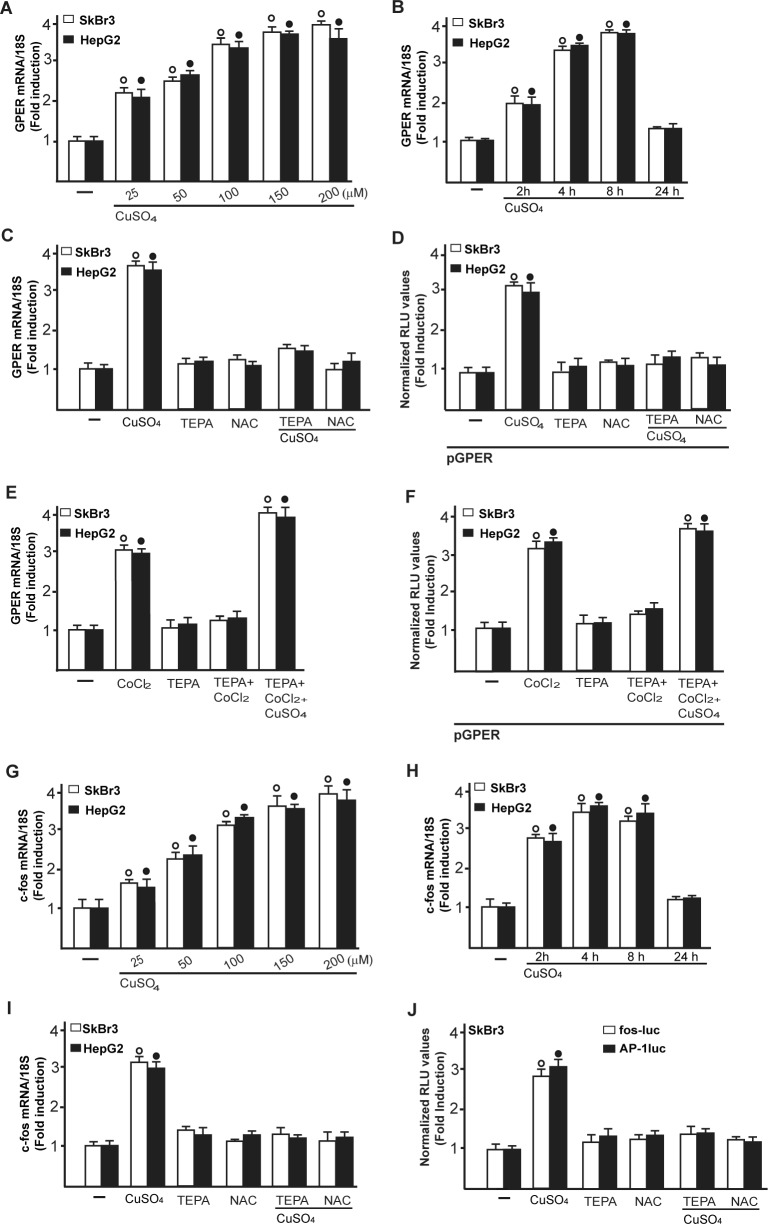
CuSO_4_ induces the mRNA expression of GPER mRNA expression of GPER in SkBr3 and HepG2 cells treated with increasing concentrations of CuSO_4_ for 8 hours, as evaluated by real-time PCR **A.** CuSO_4_ (200 μM) induces the mRNA expression of GPER in a time-dependent manner **B.** The increase in GPER mRNA observed treating SkBr3 and HepG2 cells for 8 hours with 200 μM CuSO_4_ is abrogated in the presence of TEPA (50 μM) and NAC (300 μM) **C.** The transactivation of a GPER promoter plasmid (pGPER) observed in SkBr3 and HepG2 cells treated with 200 μM CuSO_4_ for 12 hours is prevented by TEPA (50 μM) and NAC (300 μM) **D.** The mRNA induction of GPER observed in SkBr3 and HepG2 cells treated with 100 μM CoCl_2_ for 8 hours is abrogated in the presence of TEPA (50 μM) and rescued using CoCl_2_ (100 μM for 8 hours) in combination with 200 μM CuSO_4_, as determined by real-time PCR **E.** The transactivation of a GPER promoter plasmid (pGPER) observed in SkBr3 and HepG2 cells treated with 100 μM CoCl_2_ for 12 hours is prevented by TEPA (50 μM) and rescued using CoCl_2_ (100 μM for 12 hours) in combination with 200 μM CuSO_4_ **F.** Dose-response increase of c-fos mRNA expression in SkBr3 and HepG2 cells treated with CuSO_4_ for 8 hours, as evaluated by real-time PCR **G.** CuSO_4_ (200 μM) induces the mRNA expression of c-fos in a time-dependent manner **H.** The mRNA increase of c-fos observed treating SkBr3 and HepG2 cells for 8 hours with 200 μM CuSO_4_ is abrogated in the presence of TEPA (50 μM) and NAC (300 μM) **I.** The transactivation of c-fos (fos-luc) and AP-1 (AP-1luc) reporter plasmids observed in SkBr3 cells treated with 200 μM CuSO_4_ for 12 hours is prevented by TEPA (50 μM) and NAC (300 μM) **J.** In transfection assays, the luciferase activities were normalized to the internal transfection control and values of cells receiving vehicle (−) were set as 1-fold induction upon which the activities induced by treatments were calculated. In RNA experiments, values are normalized to the 18S expression and shown as fold changes of mRNA expression induced by treatments compared to cells treated with vehicle (−). Each data point represents the mean ± SD of three independent experiments performed in triplicate. (○), (●) *p* < 0.05 for cells receiving vehicle (−) *versus* treatments.

Collectively, these findings suggest that CuSO_4_ may be involved in the activation of HIF-1α/VEGF transduction signalling in cancer cells. On the basis of our recent findings suggesting that a functional cross-talk between HIF-1α and GPER may occur toward the VEGF expression in hypoxic conditions [24, 17-18], we next determined that the up-regulation of GPER mRNA expression induced by CuSO_4_ in SkBr3 and HepG2 cells (Figure 3A, 3B) is abolished in the presence of both TEPA and NAC (Figure 3C). Moreover, the transactivation of a GPER promoter construct triggered by CuSO_4_ was prevented using TEPA and NAC (Figure 3D). Notably, the GPER mRNA induction and the GPER promoter transactivation induced by CoCl_2_ were prevented in the presence of TEPA and rescued adding CuSO_4_ (Figure 3E, 3F). Results comparable to those observed upon CoCl_2_ treatment were obtained culturing cells in a low oxygen tension (2 % O_2_) (Supplementary Figure 1D-1E). Cumulatively, these data recall previous studies showing that the inhibitory effects of TEPA on hypoxia-induced responses are rescued by CuSO_4_ in a dose-dependent manner [14].

Altogether, these data indicate that GPER may be included among the transduction mediators triggered by copper, in particular in stressful conditions characterized by a low oxygen tension in cancer cells.

### Molecular mechanisms involved in the stimulatory actions elicited by CuSO_4_

**Figure 4:**
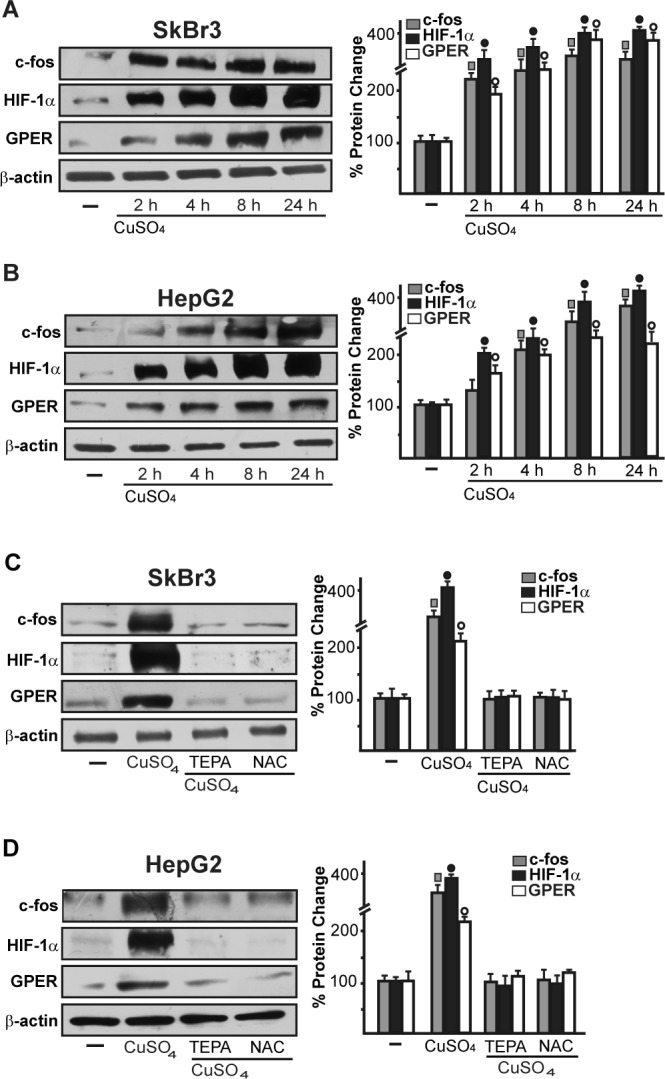
CuSO_4_ induces the protein expression of c-fos, HIF-1α and GPER Up-regulation of c-fos, HIF-1α and GPER protein expression in SkBr3 and HepG2 cells treated with 200 μM CuSO_4_ for 8 hours **A.**, **B.** The induction of c-fos, HIF-1α and GPER protein expression observed upon treatment with 200 μM CuSO_4_ for 8 hours is abolished in the presence of TEPA (50 μM) and NAC (300 μM) **C.**, **D.** Results shown are representative of three independent experiments. Side panels show densitometric analysis of the blots normalized to β-actin. (▩), (●), (○), *p* < 0.05 for cells receiving vehicle (−) *versus* CuSO_4_ treatment.

**Figure 5:**
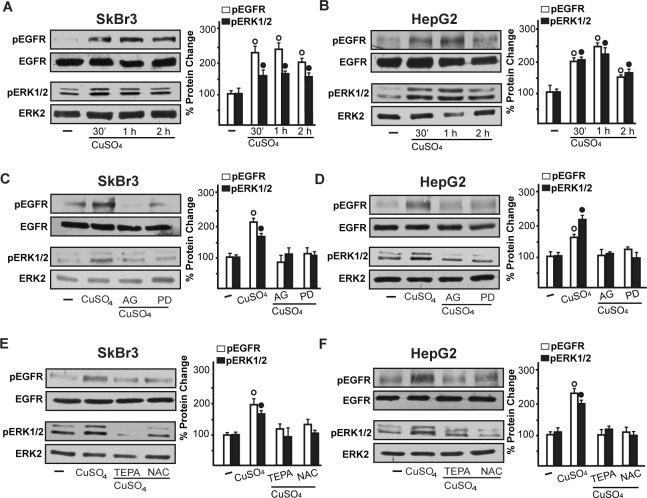
CuSO_4_ induces EGFR and ERK activation The exposure to 200 μM CuSO_4_ induces EGFR (Tyr 1173) and ERK1/2 phosphorylation in SkBr3 and HepG2 cells **A.**, **B.** The activation of EGFR and ERK1/2 observed in SkBr3 and HepG2 cells treated with 200 μM CuSO_4_ for 30 min is abrogated in the presence of the EGFR inhibitor AG1478 (AG, 10 μM) and the MEK inhibitor PD98059 (PD, 10 μM) **C.**, **D.** as well as TEPA (50 μM) and NAC (300 μM) **E.**, **F.** Side panels show densitometric analysis of the blots normalized to EGFR or ERK2. Each data point represents the mean ± SD of three independent experiments. (○), (●) *p* < 0.05 for cells receiving vehicle (−) *versus* CuSO_4_ treatment.

**Figure 6:**
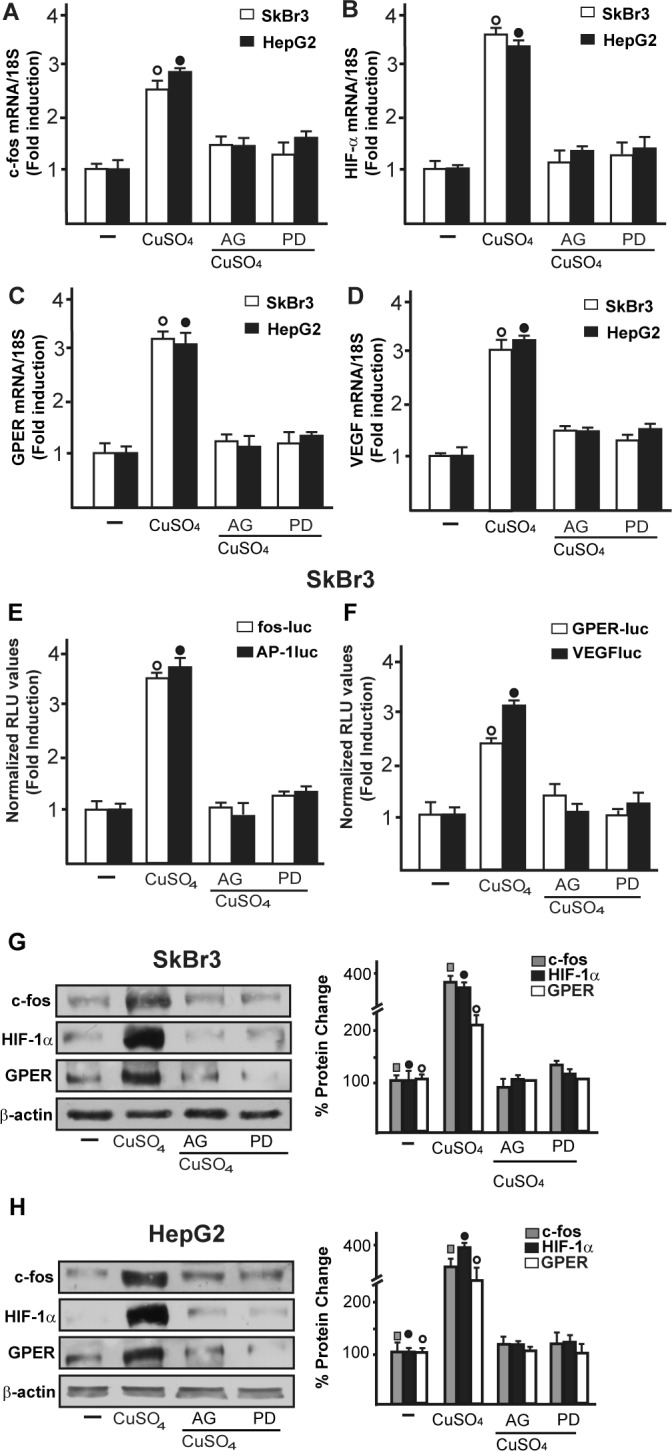
The EGFR/ERK transduction pathway is involved in the stimulatory responses induced by CuSO_4_ The mRNA increase of c-fos **A.**, HIF-1α **B.**, GPER **C.** and VEGF **D.** observed in SkBr3 and HepG2 cells upon treatment with 200 μM CuSO_4_ for 8 hours is prevented by AG (10 μM) and PD (10 μM), as evaluated by real-time PCR. Values are normalized to the 18S expression and shown as fold changes of mRNA expression induced by CuSO_4_ compared to cells treated with vehicle (−). The transactivation of c-fos, AP-1, GPER and VEGF reporter plasmids induced in SkBr3 cells upon treatment with 200 μM CuSO_4_ for 12 hours is abolished using AG (10 μM) and PD (10 μM) **E.**, **F.** The luciferase activities were normalized to the internal transfection control and values of cells receiving vehicle (−) were set as 1-fold induction upon which the activities induced by CuSO_4_ treatment were calculated. Each data point represents the mean ± SD of three independent experiments performed in triplicate. The up-regulation of c-fos, HIF-1α and GPER protein expression observed in SkBr3 **G.** and HepG2 **H.** cells treated with 200 μM CuSO_4_ for 8 hours is abolished in the presence of AG (10 μM) and PD (10 μM) **G.**, **H.** Results shown are representative of three independent experiments. Side panels show densitometric analysis of the blots normalized to β-actin. (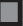), (●), (○), *p* < 0.05 for cells receiving vehicle (−) *versus* CuSO_4_ treatment.

As c-fos expression is a molecular sensor of both GPER and HIF-1α signalling [18, 25-26], we also demonstrated that c-fos mRNA increase upon CuSO_4_ stimulation (Figure 3G, 3H) is abrogated in the presence of TEPA and NAC (Figure 3I). Nicely fitting with these results, the transactivation of a c-fos luciferase construct and AP1-luc promoter sequence induced by CuSO_4_ was repressed in the presence of TEPA and NAC (Figure 3J). Recapitulating the aforementioned findings, the protein induction of c-fos, HIF-1α and GPER observed upon CuSO_4_ treatment was abrogated in the presence of TEPA and NAC in SkBr3 and HepG2 cells (Figure 4A-4D). Given that the activation of EGFR/ERK signalling triggers transduction mechanisms leading to gene expression changes as mentioned above [17-18, 25, 27-28], we ascertained that the EGFR and ERK1/2 phosphorylation induced by CuSO_4_ in both SkBr3 and HepG2 cells (Figure 5A, 5B) is blocked in the presence of the EGFR tyrosine kinase inhibitor AG1478 (AG) and the MEK inhibitor PD98059 (PD) (Figure 5C, 5D) as well as using TEPA and NAC (Figure 5E, 5F). Further corroborating these data, the up-regulation of c-fos, HIF-1α, GPER and VEGF mRNA expression (Figure 6A-6D) as well as the transactivation of fos-luc, AP1-luc, GPER-luc and VEGF-luc reporter constructs (Figure 6E, 6F) induced by CuSO_4_ were abolished in the presence of AG and PD. Analogous findings were obtained evaluating the regulation of c-fos, HIF-1α and GPER protein expression in SkBr3 and HepG2 cells (Figure 6G, 6H).

**Figure 7:**
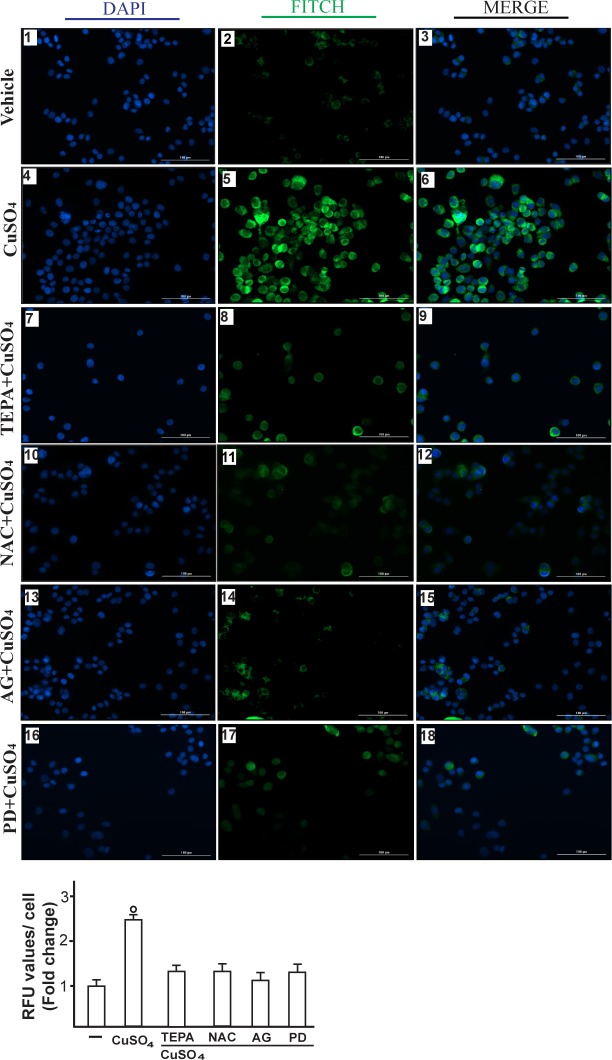
CuSO_4_ induces VEGF protein expression as evaluated by immunofluorescence assay SkBr3 cells were treated for 12 hours with vehicle (panels 1-3), 200 μM CuSO_4_ alone (panels 4-6) or in combination with TEPA (50 μM) (panels 7-9), NAC (300 μM) (panels 10-12), AG (10 μM) (panels 13-15) and PD (10 μM) (panels 16-18). VEGF accumulation is shown by the green signal, nuclei were stained by DAPI (blue signal). The slides were imaged on the Cytation 3 Cell Imaging Multimode Reader (BioTek, Winooski, VT). Images shown are representative of three independent experiments. Fluorescence intensities for the green channel were quantified in 10 random fields for each condition and results are expressed as fold change of relative fluorescence units (RFU) over the vehicle-treated cells (as indicated in the lower panel). Values are mean ± SD of three independent experiments. (○) *p* < 0.05 for cells receiving vehicle (−) *versus* CuSO_4_ treatment.

**Figure 8:**
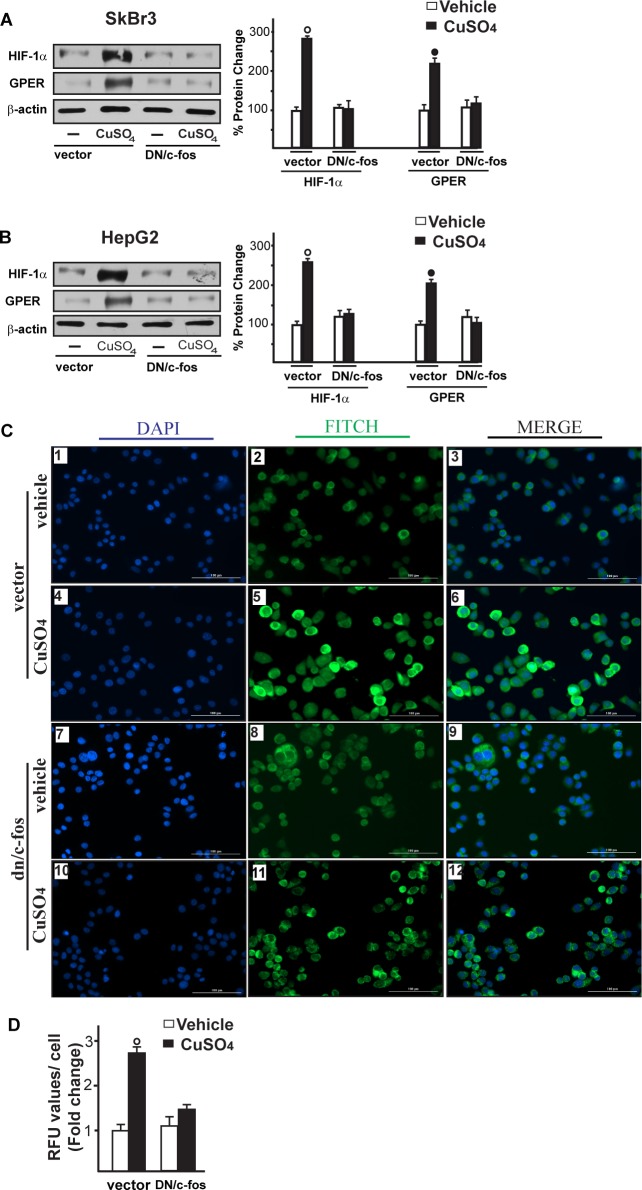
c-fos is involved in the up-regulation of HIF-1α, GPER and VEGF induced by CuSO_4_ Evaluation of HIF-1α and GPER protein expression in SkBr3 and HepG2 cells transfected for 24 hours with a vector or a plasmid encoding for a dominant negative form of c-fos (DN/c-fos) and then treated with 200 μM CuSO_4_ for 8 hours (**A**., **B**.). Side panels show densitometric analysis of the blots normalized to β-actin. Each data point represents the mean ± SD of three independent experiments. Evaluation of VEGF protein expression by immunofluorescence assay in SkBr3 cells transfected for 24 hours with a vector (panels 1-6) or a plasmid encoding for a dominant negative form of c-fos (DN/c-fos) (panels 7-12) and then treated with vehicle or 200 μM CuSO_4_ for 12 hours, as indicated. VEGF accumulation is shown by the green signal, nuclei were stained by DAPI (blue signal). The slides were imaged on the Cytation 3 Cell Imaging Multimode Reader (BioTek, Winooski, VT). Images shown are representative of three independent experiments **C.** Fluorescence intensities for the green channel were quantified in 10 random fields for each condition and results are expressed as fold change of relative fluorescence units (RFU) over the vehicle-treated cells **D.** Values are mean ± SD of three independent experiments. (○), (●) *p* < 0.05 for cells receiving vehicle (−) *versus* CuSO_4_ treatment.

**Figure 9:**
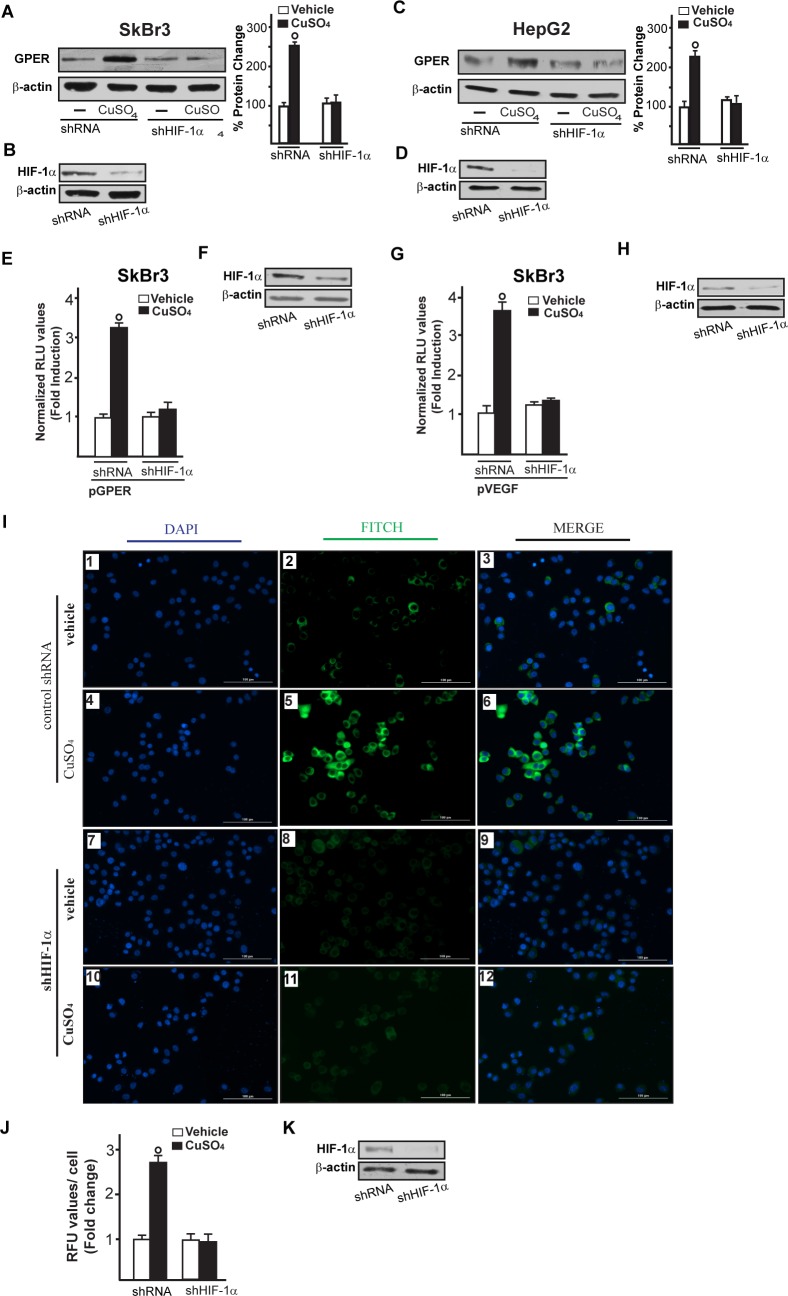
HIF-1α is involved in the up-regulation of GPER and VEGF induced by CuSO_4_ Evaluation of GPER protein expression in SkBr3 and HepG2 cells transfected with shRNA or shHIF-1α for 24 hours and then treated with 200 μM CuSO_4_ for 8 hours **A.**, **C.** Side panels show densitometric analysis of the blots normalized to β-actin. Efficacy of HIF-1α silencing in SkBr3 and HepG2. Each data point represents the mean ± SD of three independent experiments **B.**, **D. E.**-**H.** The transactivation of the GPER (pGPER) **E.** and VEGF (pVEGF) **G.** promoter plasmids observed in SkBr3 cells treated with 200 μM CuSO_4_ for 12 hours is abrogated silencing the expression of HIF-1α. (F, H) Efficacy of HIF-1α silencing. The luciferase activities were normalized to the internal transfection control and values of cells receiving vehicle were set as 1-fold induction, upon which the activities induced by treatments were calculated. Each data point represents the mean ± SD of three independent experiments performed in triplicate. **I.** Evaluation of VEGF protein expression by immunofluorescence assay in SkBr3 cells transfected for 24 hours with shRNA (panels 1-6) or shHIF-1α (panels 7-12) and treated with 200 μM CuSO_4_ for 12 hours, as indicated. VEGF accumulation is shown by the green signal, nuclei were stained by DAPI (blue signal). The slides were imaged on the Cytation 3 Cell Imaging Multimode Reader (BioTek, Winooski, VT). Images shown are representative of three independent experiments. **J.** Fluorescence intensities for the green channel were quantified in 10 random fields for each condition and results are expressed as fold change of relative fluorescence units (RFU) over the vehicle-treated cells. Values are mean ± SD of three independent experiments. **K.** Efficacy of HIF-1α silencing. (○) *p* < 0.05 for cells receiving vehicle *versus* CuSO_4_ treatment.

**Figure 10:**
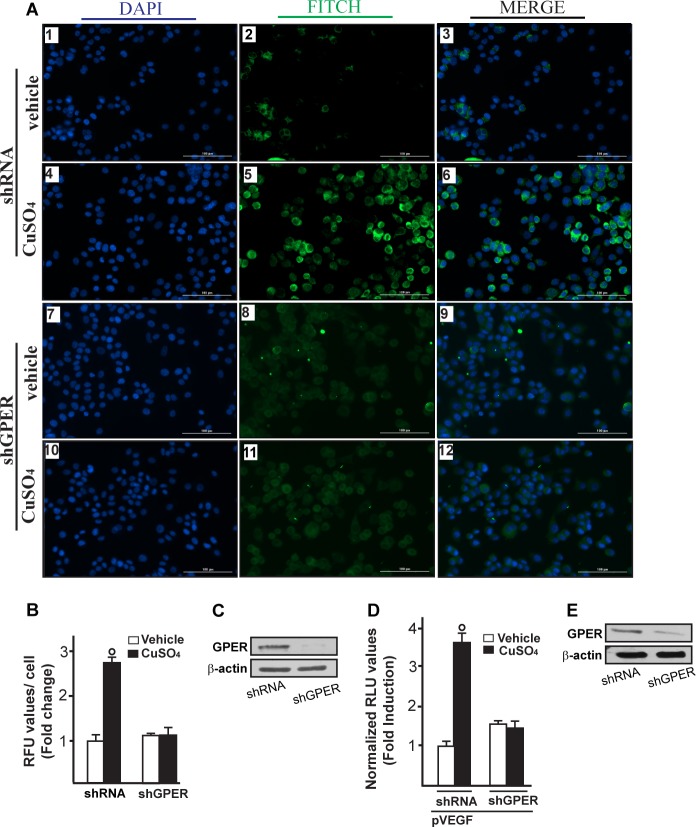
GPER is involved in VEGF protein increase induced by CuSO_4_ Evaluation of VEGF protein expression by immunofluorescence assay in SkBr3 cells transfected for 24 hours with shRNA (panels 1-6) or shGPER (panels 7-12) and treated with 200 μM CuSO_4_ for 12 hours, as indicated. VEGF accumulation is evidenced by the green signal, nuclei were stained by DAPI (blue signal). The slides were imaged on the Cytation 3 Cell Imaging Multimode Reader (BioTek, Winooski, VT). Images shown are representative of three independent experiments **A.** Fluorescence intensities for the green channel were quantified in 10 random fields for each condition and results are expressed as fold change of relative fluorescence units (RFU) over the vehicle-treated cells **B.** Values are mean ± SD of three independent experiments. Efficacy of GPER silencing **C.** The transactivation of the VEGF (pVEGF) promoter plasmid observed in SkBr3 cells treated with 200 μM CuSO_4_ for 12 hours is abrogated silencing the expression of GPER **D.** The luciferase activities were normalized to the internal transfection control and values of cells receiving vehicle were set as 1-fold induction, upon which the activities induced by treatments were calculated. Efficacy of GPER silencing **E.** Each data point represents the mean ± SD of three independent experiments performed in triplicate. (○) *p* < 0.05 for cells receiving vehicle (−) *versus* CuSO_4_ treatment.

Immunofluorescence experiments performed in SkBr3 cells showed that TEPA, NAC, AG and PD prevent also the increase of VEGF protein expression upon CuSO_4_ treatment (Figure 7). In addition, the HIF-1α protein increase triggered by CuSO_4_ was no longer evident transfecting SkBr3 and HepG2 cells with a plasmid encoding a dominant/negative c-fos mutant (DN/c-fos) (Figure 8A, 8B). In accordance with the aforementioned results, the up-regulation of GPER (Figure 8A, 8B) and VEGF (Figure 8C, 8D) protein levels upon CuSO_4_ treatment was prevented by DN/c-fos, as evaluated by immunoblotting and immunofluorescence assays, respectively. As demonstrated in our previous investigations, in hypoxic tumor microenvironment HIF-1α mediates the expression of GPER that contributes to the regulation and function of VEGF [17, 24]. Likewise, we found that the GPER protein up-regulation induced by CuSO_4_ as well as the transactivation of the GPER promoter were abolished knocking down HIF-1α expression (Figure 9A-9F). In addition, the silencing of HIF-1α prevented the CuSO_4_ -induced activation of the VEGF promoter construct (Figure 9G, 9H) as well as the up-regulation of VEGF protein expression (Figure 9I-9K). Of note, GPER was required for VEGF protein induction and the transactivation of a VEGF promoter construct by CuSO_4_, as demonstrated by silencing experiments (Figure 10A-10E). Overall, these data highlight the transduction mechanisms involved by copper toward the stimulation of VEGF in cancer cells.

### HIF-1α and GPER are required for VEGF-induced endothelial tube formation, cell migration and proliferation induced by CuSO_4_

**Figure 11:**
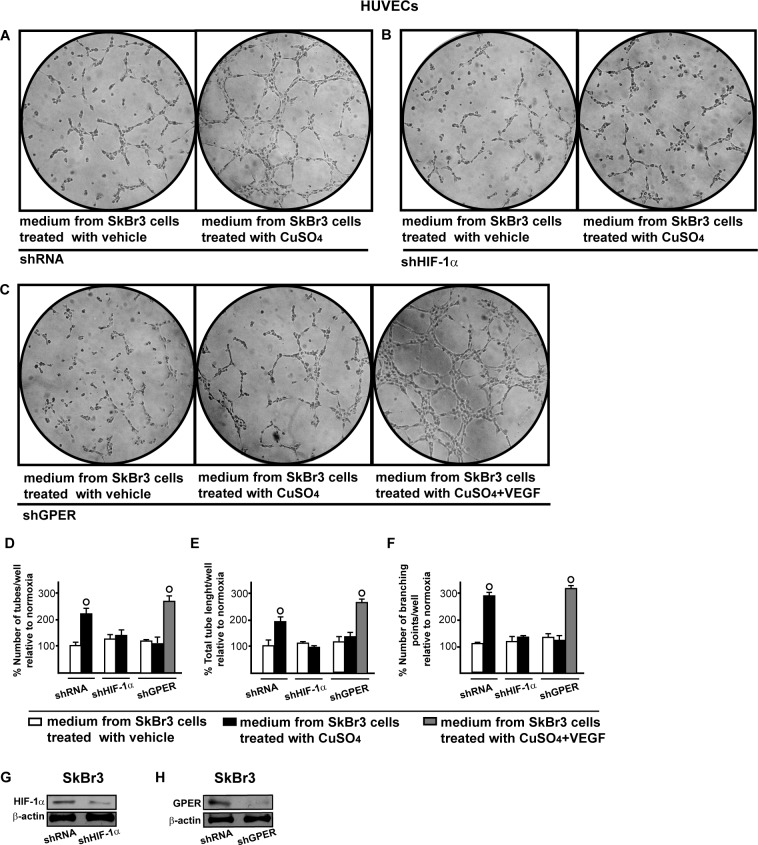
HIF-1α and GPER contribute to the endothelial tube formation triggered by CuSO_4_ Tube formation in HUVECs cultured for 2 hours in medium collected from SkBr3 cells which were transfected for 24 hours with shRNA **A.**, shHIF-1α **B.** or shGPER **C.** and then treated for 18 hours with vehicle or 200 μM CuSO_4_, as indicated. **C.** In HUVECs cultured in conditioned medium from SkBr3 cells that were transfected with shGPER and treated with 200 μM CuSO_4,_ tube formation is rescued adding 10 ng/mL VEGF for 2 hours. Data are representative of three independent experiments performed in triplicate. Quantification of the number of tubes **D.**, total tube length **E.** and number of branching points **F.** observed in HUVECs, as indicated. Data are representative of three independent experiments performed in triplicate. (○) *p* < 0.05 for cells receiving medium from SkBr3 cells treated with vehicle *versus* cells receiving medium from SkBr3 cells treated with CuSO_4_. Efficacy of HIF-1α **G.** and GPER **H.** silencing in SkBr3 cells.

**Figure 12:**
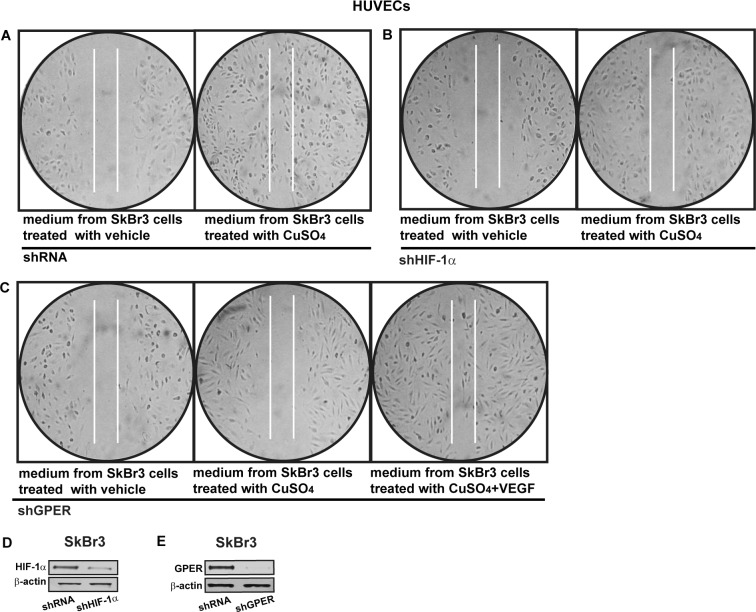
HIF-1α and GPER contribute to the endothelial cell migration induced by CuSO_4_ Cell migration in HUVECs cultured for 24 hours in medium collected from SkBr3 cells which were transfected for 24 hours with control shRNA **A.**, shHIF-1α **B.** or shGPER **C.** and then treated for 18 hours with vehicle or 200 μM CuSO_4_, as indicated. **C.** In HUVECs cultured in medium from SkBr3 cells which were transfected with shGPER and treated with 200 μM CuSO_4,_ cell migration is rescued adding 10 ng/mL VEGF for 36 hours. Data are representative of three independent experiments performed in triplicate. Efficacy of HIF-1α **D.** and GPER **E.** silencing in SkBr3 cells.

Having established that HIF-1α and GPER cooperate in triggering the up-regulation of VEGF by CuSO_4_, we assessed in HUVECs the involvement of HIF-1α and GPER in the formation of tubule-like structures that represent a useful experimental model of angiogenic process [29]. Interestingly, a ramified network of tubules was generated in HUVECs cultured in conditioned medium from CuSO_4_ -treated SkBr3 cells (Figure 11A). However this effect was prevented by knocking down the expression of HIF-1α or GPER (Figure 11B-11H). The addition of VEGF to the medium collected from CuSO_4_ -treated and GPER-silenced SkBr3 cells rescued the generation of tubule structures in HUVECs (Figure 11C). Figure 11 (panels D-F) recapitulates these results, suggesting that VEGF may be considered as a target of copper-activated HIF-1α/GPER signalling toward new blood vessels formation. As in previous studies VEGF boosted endothelial cells migration [30-31] we then evaluated whether HIF-1α and GPER are involved in the migration of HUVECs. Conditioned medium from SkBr3 cells exposed to CuSO_4_ induced the migration of HUVECs (Figure 12A), however this response was abrogated silencing HIF-1α and GPER expression (Figure 12B-12E). Indeed, the addition of VEGF rescued cell migration culturing HUVECs in medium collected from SkBr3 cells which were GPER-silenced and treated with CuSO_4_ (Figure 12C). Next, we determined that HIF-1α and GPER are required for SkBr3 cell proliferation induced by CuSO_4_, as this response was prevented knocking-down their expression (Supplementary Figure S2A-C). Likewise, the growth effects elicited by CuSO_4_ was abolished in the presence of TEPA (Supplementary Figure S2D). Altogether, these findings suggest that copper may trigger relevant biological actions through HIF-1α/GPER/VEGF transduction signalling in both cancer and endothelial cells toward angiogenesis and tumor progression.

## DISCUSSION

The present study provides novel evidence regarding the molecular mechanisms by which copper may trigger the expression and function of VEGF toward angiogenesis and tumor progression. In particular, we have shown that copper activates the EGFR/ERK/c-fos transduction pathway leading to the expression of HIF-1α, GPER and VEGF in breast and hepatic cancer cells. In this regard, we demonstrated that a functional cooperation between HIF-1α and GPER contributes to VEGF regulation in cancer cells exposed to copper. Recalling previous studies on the capability of copper chelating agents to elicit anti-tumor effects [5, 32], we have also evidenced that these chemicals exert an inhibitory action on HIF-1α/GPER/VEGF transduction pathway. Next, we have found that HIF-1α and GPER are required for endothelial tube formation and cell migration stimulated by VEGF as well as for copper-induced proliferation of breast cancer cells.

The role of copper in tumor initiation and progression has been extensively investigated both *in vitro* and *in vivo* [8, 33]. In this context, high copper levels ranging from 50 to 200μM have been correlated with incidence and recurrence in cancer patients [8, 9]. In accordance with these findings, we have ascertained that copper exerts stimulatory effects on gene expression starting from a concentration of 25 μM, even though the maximal responses were observed using a concentration of 200 μM. Hence, the last amount was used in all assays to better evaluate the potential of copper to activate the aforementioned biological activity. Previous studies have disclosed that certain effects elicited by copper in cancer cells rely on the generation of reactive oxygen species (ROS), which act as second messenger in triggering stimulatory signals [8]. In this regard, it has been shown the transduction mechanisms involved, that include the activation of the EGFR/ERK pathway and the expression of genes mediating growth responses like c-fos [reviewed in 5]. On the basis of these observations, it could be argued that copper may mimic some biological features which characterize the hypoxic tumor environment.

HIF-1 acts as a survival factor upon low oxygen conditions regulating the expression of genes involved in cell metabolism, migration, invasion and angiogenesis [34-35]. In this vein, it is worth mentioning that copper was shown to increase HIF-1α stabilization and accumulation [19]. Further extending these findings, our current results indicate that copper is also able to induce HIF-1α expression, thus providing a new mechanism through which this chemical may be involved in cancer progression. Previous studies have determined that GPER contributes together with HIF-1α to the adaptive responses to hypoxic tumor microenvironment [17, 24]. Nicely fitting with these observations, the present data reveal that copper induces the expression of GPER through HIF-1α, leading to the regulation of VEGF in breast cancer cells and cancer associated fibroblasts (CAFs) [17]. The stimulatory role of copper in cancer development has been also proved by copper chelating agents as a reduction in tumor volume, vascular permeability, tumor's microvascular supply and micrometastasis generation has been reported lowering copper levels in diverse experimental models [5]. Extending the current knowledge on the action of anti-copper drugs like TEPA, our data indicate that these chemicals may also target HIF-1α/GPER signalling among the multifaceted responses triggered in cancer cells.

To date, the expression of GPER has been associated with negative clinical features and poor survival rates in a variety of tumors [36-38]. Consequently, huge efforts are currently underway to better understand the mechanisms involved in the regulation of GPER [28, 39-58] which belongs to the GPCRs family widely involved in cancer progression [59, 60]. Of note, several studies have demonstrated that estrogenic GPER signalling mediates relevant biological effects like proliferation and migration in cancer cells and CAFs [61-63] that are largely acknowledged to contribute to tumor cell metabolism and disease progression [64-66]. In this regard, additional investigations are needed to determine whether copper could be also able to activate GPER signalling in a direct manner, as previously demonstrated using other metals [67].

Here, we have provided novel evidence regarding the action elicited by copper toward tumor angiogenesis and progression. On the basis of the present findings GPER may be included together with HIF-1α and VEGF among the molecular targets of copper chelating agents in combination therapies. Nevertheless, further studies are needed to better define the role of copper on the functional interaction between GPER, HIF-1α and VEGF in malignant cells and tumor microenvironment.

## MATERIALS AND METHODS

### Materials

Copper sulfate (CuSO_4_), cobalt chloride (CoCl_2_), tetraethylenepentamine (TEPA) and ROS scavenger N-acetyl-L-cysteine (NAC) were purchased from Sigma-Aldrich Srl (Milan, Italy). Tyrphostin AG1478 (AG) was purchased from Biomol Research Laboratories, Inc (Milan, Italy). PD98059 (PD) was obtained from Calbiochem (Milan, Italy). Human VEGF was purchased from Peprotech (Rocky Hill, New Jersey, USA). All compounds were dissolved in DMSO, except VEGF, CuSO_4_ and NAC which were solubilized in water.

### Cell cultures

We used SkBr3 breast cancer cells and HepG2 hepatocarcinoma cells that represent a valuable tool for the evaluation of the transduction pathways activated by copper in cancer cells. As both cell lines express GPER, which has been involved with the angiogenic process within the tumor microenvironment [17-18], this model system is suitable to ascertain the contribution of GPER to copper action toward tumor angiogenesis.

The SkBr3 breast cancer cells were maintained in RPMI-1640 (Life Technologies, Milan, Italy) without phenol red, supplemented with 10% fetal bovine serum (FBS) and 100 μg/ml penicillin/streptomycin. The hepatocarcinoma cells HepG2 were cultured in DMEM (Dulbecco's modified Eagle's medium) (Life Technologies, Milan, Italy) with phenol red, supplemented with 10% FBS and 100 μg/ml penicillin/streptomycin. Human umbilical vein endothelial cells (HUVECs) were seeded on collagen-coated flasks (Sigma-Aldrich Srl, Milan, Italy) and cultured in Endothelial Growth Medium (EGM) (Lonza, Milan, Italy), supplemented with 5% FBS (Lonza, Milan, Italy). All cell lines were grown in a 37° C HeraCell incubator (ThermoScientific-Heraeus, Milan, Italy) with 5% CO_2_. For hypoxic stimulation, cells were treated with CoCl_2_ (100 μM) or cultured in the presence of a low oxygen tension (2% O_2_) in a multi-gas HeraCell incubator (ThermoScientific-Heraeus, Milan, Italy). Cells were switched to medium without serum the day before experiments.

### Gene reporter assays

The 2.6 kb VEGF promoter-luciferase construct containing full-length VEGF promoter sequence (22,361 to +298 bp relative to the transcription start site) used in luciferase assays was a kind gift from dr. P. Soumitro (Harvard Medical School, Boston, Massachusetts). The GPER promoter-luciferase construct (pGPER 2.9 kb) was obtained as previously described [24].

The luciferase reporter plasmid for AP-1 responsive collagen promoter was a kind gift from H. Van Dam (Department of Molecular Cell Biology, Leiden University, Leiden, Netherlands). The luciferase reporter plasmid for c-fos, encoding a −2.2 kb 5′ upstream fragment of human c-fos, kindly provided by K. Nose (Department of Microbiology, Showa University School of Pharmaceutical Sciences, Hatanodai, Shinagawa-ku, Tokyo, Japan). SkBr3 and HepG2 cells (1 × 10^5^) were plated into 24-well dishes with 500μL/well culture medium containing 10% FBS. Transfections were performed using X-treme GENE 9 DNA transfection reagent as recommended by the manufacturer (Roche Diagnostics, Milan, Italy), with a mixture containing 0.5μg of reporter plasmid and 10 ng of pRL-TK. After 24 h, cells were treated with CuSO_4_, alone and in combination with TEPA, NAC, AG1478 and PD98059, as indicated. For co-transfection experiments, cells were previously transfected with control shRNA, shHIF-1α or shGPER using X-treme GENE 9 DNA transfection reagent (Roche Diagnostics, Milan, Italy). A mixture containing 0.5 μg of reporter plasmid and 10 ng of pRL-TK was then transfected by using X-treme GENE 9 DNA Transfection. After 8 hours, cells were treated for 18 hours with CuSO_4_ in serum free medium. Luciferase activity was measured with the Dual Luciferase Kit (Promega, Milan, Italy) normalized to the internal transfection control provided by Renilla luciferase activity. The normalized relative light unit values obtained from cells treated with vehicle were set as 1-fold induction, upon which the activity induced by treatments was calculated.

### Gene expression studies

Total RNA was extracted from cell cultures using the TRIzol commercial kit (Life Technologies, Milan, Italy) according to the manufacturer's protocol. RNA was quantified spectrophotometrically and quality was checked by electrophoresis through agarose gels stained with ethidium bromide. Only samples that were not degraded and showed clear 18 and 28 S bands under UV light were used for RT-PCR. Total cDNA was synthesized from the RNA by reverse transcription as previously described [17]. The expression of selected genes was quantified by real-time PCR using Step One ^(TM)^ sequence detection system (Applied Biosystems Inc, Milan, Italy), following the manufacturer's instructions. Gene-specific primers were designed using Primer Express version 2.0 software (Applied Biosystems. Inc., Milan, Italy) and are as follows: HIF-1α Fwd: 5′-TGCATCTCCATCTTCTACCCAAGT-3′ and Rev: 5′-CCGACTGTGAGTGCCACTGT-3′; VEGF Fwd: 5′- TGCAGATTATGCGGATCAAACC-3′ and Rev: 5′- TGCATTCACATTTGTTGTGCTGTAG-3′; GPER Fwd: 5′-CCTGGACGAGCAGTATTACGATATC-3′ and Rev 5′-TGCTGTACATGTTGATCTG-3′; c-FOS Fwd: 5′-GAGCCCTTTGATGACTTCCT-3′ and Rev: 5′-GAGCGGGCTGTCTCAGA-3′; 18S Fwd: 5′- GGCGTCCCCCAACTTCTTA -3′ and Rev: 5′- GGGCATCACAGACCTGTTATT -3′. Assays were performed in triplicate and the results were normalized for 18S expression and then calculated as fold induction of RNA expression.

### Western blot analysis

SkBr3 and HepG2 cells were processed according to the previously described protocol [17] to obtain protein lysate that was electrophoresed through a reducing SDS/10% (w/v) polyacrylamide gel, electroblotted onto a nitrocellulose membrane and probed with primary antibodies against HIF-1α (R&D Systems, Inc. Celbio, Milan, Italy), GPER (N-15), c-fos (H-125), phosphorylated ERK 1/2 (E-4), ERK2 (C-14), EGFR (1005), pEGFR Tyr 1173 (sc-12351-R) and β-actin (C2), all purchased from Santa Cruz Biotechnology, (DBA, Milan, Italy). Proteins were detected by horseradish peroxidase-linked secondary antibodies (Santa Cruz Biotecnology, DBA) and revealed using the ECL System (GE Healthcare).

### Gene silencing experiments

Cells were plated onto 10-cm dishes and prior to treatments cells were transfected for 24 hours using X-treme GENE 9 DNA Transfection Reagent (Roche Diagnostics, Milan, Italy) with a control shRNA, shHIF-1α, shGPER, a control vector and the plasmid DN/c-fos, encoding a c-fos mutant that heterodimerizes with c-fos dimerization partners but not allowing DNA binding (kindly obtained from Dr. C. Vinson, NIH, Bethesda, MD, USA). The HIF-1α shRNA and the respective control plasmid were purchased from SABioscience Corporation (Frederick, MD, USA). The silencing of GPER expression was obtained by the construct which we have previously described and used [68].

### Immunofluorescence assay

Fifty percent confluent cultured SkBr3 cells grown on coverslips were serum deprived and then treated for 12 hours with CuSO_4_ alone and in combination with TEPA, NAC, AG1478 and PD98059, as indicated. Where required, cells previously transfected for 24 h with shHIF-1α or shGPER and respective negative control plasmids (as described above) and then treated for 18 hours with CuSO_4,_ Then cells were fixed in 4% paraformaldehyde, permeabilized with 0.2% Triton X-100, washed three times with PBS and incubated overnight with a mouse primary antibody against VEGF (C-1) (Santa Cruz Biotechnology, DBA, Milan, Italy). After incubation, the slides were extensively washed with PBS and incubated with 4′,6-diamidino-2-phenylindole dihydrochloride (DAPI), (1:1000), (Sigma-Aldrich, Milan, Italy) and donkey anti-mouse IgG-FITC (1:300; purchased from Alexa Fluor, Life Technologies, Milan, Italy). The slides were imaged on the Cytation 3 Cell Imaging Multimode reader (BioTek, Winooski, VT) and analysed using the software Gen5 (BioTek, Winooski, VT).

### Conditioned medium

SkBr3 cells were cultured in regular growth medium, then cells were washed twice with PBS and transfected for 24 hours in serum-free RPMI-1640 with shHIF-1α, shGPER or control shRNA using X-treme GENE 9 DNA Transfection Reagent (Roche Diagnostics, Milan, Italy). Cells were treated for 18 hours with CuSO_4_, culture medium was then replaced for additional 18 hours with medium without serum. Thereafter, the supernatants were collected, centrifuged at 3,500 rpm for 5 minutes to remove cell debris and used as conditioned medium in HUVECs.

### Tube formation assay

The day before the experiment, confluent HUVECs were starved overnight at 37°C in serum free medium (EBM, Lonza, Milan, Italy). Growth factor-reduced Matrigel® (Cultrex, Trevigen Inc, USA) was thawed overnight at 4°C on ice, plated on the bottom of prechilled 96well-plates and left at 37°C for 1 h for gelification. Starved HUVECs were collected by enzymatic detachment (0.25% trypsin-EDTA solution, Life Technologies, Milan, Italy), counted and resuspended in conditioned medium from CAFs. Then, 10,000 cells/well were seeded on Matrigel and incubated at 37°C. Tube formation was observed starting from 2 h after cell seeding and quantified by using the software NIH ImageJ (National Institutes of Health (NIH), Rockville Pike, Bethesda, Maryland, USA).

### Migration assay

Twelve-well plates were coated with 500 μL fibronectin for 2 hours at 37°C (Sigma Aldrich, Milan, Italy). HUVECs were allowed to grow in regular growth medium until they reached a 70% to 80% confluence. Next, to create a scratch of the cell monolayer, a p200 pipette tip was used. Cells were washed twice with PBS and then incubated in medium collected from SkBr3 cells as previously described. The migration assay was evaluated after 24 hours of treatment.

### MTT growth assay

For quantitative proliferation assay, cells (1 × 10^5^) were seeded in 24-well plates in regular growth medium. Cells were washed once they had attached and then incubated in medium containing 2.5% charcoal-stripped FBS with the indicated treatments; medium was renewed every day (with treatments) before dimethylthiazoldiphenyltetrazoliumbromide (MTT, Sigma-Aldrich, Milan, Italy) assay which was performed according to the manufacturer's protocol. A concentration of 250ng/L of the control shRNA, shHIF-1α or shGPER plasmids was transfected using X-treme GENE 9 DNA Transfection Reagent the day before treatments. The absorbance was measured using a FLX-800 microplate fluorimeter (Bio-Tek Instruments, Inc., Winooski, VT, USA) at a test wavelength of 570 nm. Each experiment was performed at in triplicate.

### Statistical analysis

Statistical analysis was performed using ANOVA followed by Newman-Keuls' testing to determine differences in means. *p* < 0.05 was considered statistically significant.

## SUPPLEMENTARY MATERIAL FIGURES


