# Supplementary material for: Copper activates HIF-1α/GPER/VEGF signalling in cancer cells
Source: Oncotarget. 2015 Sep 22;6(33):34158–77. doi: 10.18632/oncotarget.5779 (PMC4741443; doi:10.18632/oncotarget.5779)
Supplement: Supplementary file 1 [file oncotarget-06-34158-s001.pdf]

Copper activates HIF-1α/GPER/VEGF signalling in cancer cells

Supplementary Material

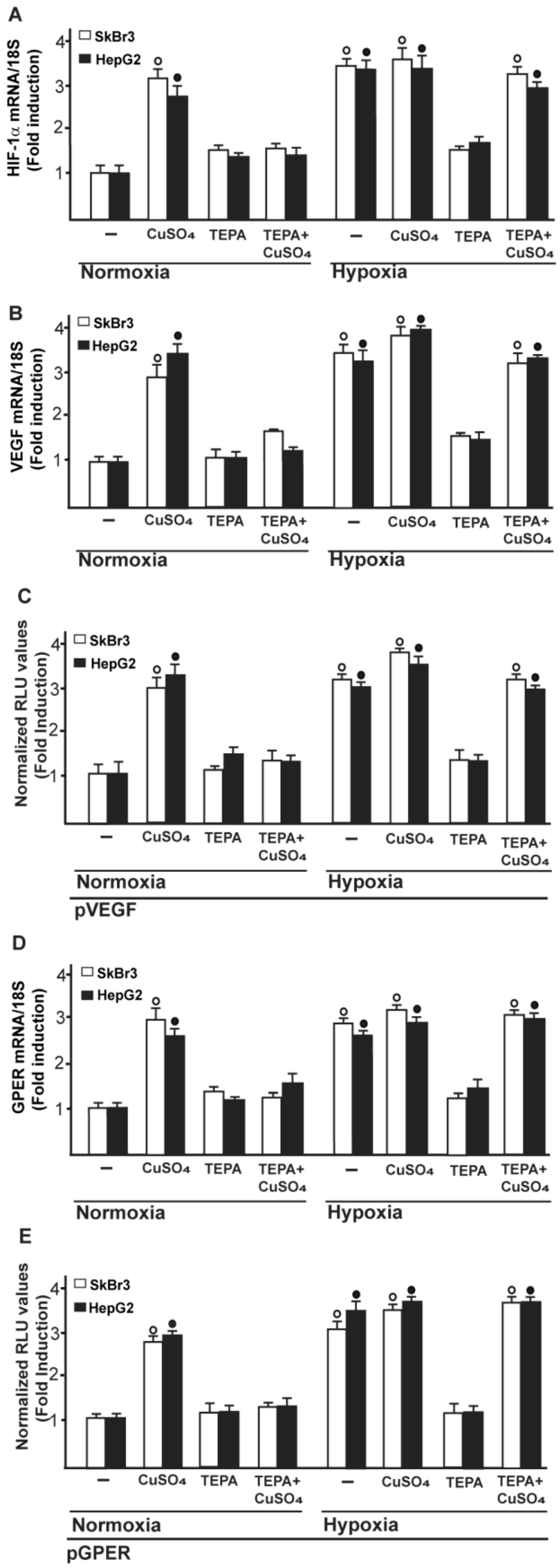

Supplementary Fig. 1: *$CuSO_4$ rescues the inhibitory effects of TEPA on hypoxia-induced transcription of HIF-1α, GPER and VEGF.* In SkBr3 and HepG2 cells, the up-regulation of HIF-1α (A) and VEGF (B) mRNA expression induced by a treatment with 200 µM $CuSO_4$ for 8 hours is no longer evident in the presence of TEPA (50 µM) in normoxic condition. The up-regulation of HIF-1α (A) and VEGF (B) mRNA expression induced by hypoxia (2% O_2 for 8 hours) is no longer evident in the presence of TEPA (50 µM) but rescued culturing cells in the aforementioned hypoxic condition along with 200 µM $CuSO_4$, as determined by real-time PCR. Values are normalized to the 18S expression and shown as fold changes of mRNA expression induced by treatments also upon hypoxia respect to cells cultured with vehicle (-) in normoxic condition. (○), (●) indicate $p < 0.05$ (C) In SkBr3 and HepG2 cells, the transactivation of a VEGF promoter plasmid (pVEGF) observed upon treatment with 200 µM $CuSO_4$ for 12 hours is no longer evident in the presence of TEPA (50 µM) in normoxic condition. The transactivation of a VEGF promoter plasmid (pVEGF) induced by hypoxia (2% O_2 for 12 hours) is no longer evident in the presence of TEPA (50 µM) but rescued culturing cells in the aforementioned hypoxic condition along with 200 µM $CuSO_4$. The luciferase activities were normalized to the internal transfection control and values of cells receiving vehicle (-) and cultured upon normoxia were set as 1-fold induction upon which the activities induced by treatments also in hypoxic conditions were calculated. Each data point represents the mean ± SD of three independent experiments performed in triplicate. (○), (●) indicate $p < 0.05$ (D) In SkBr3 and HepG2 cells, the up-regulation of GPER mRNA expression induced by a treatment with 200 µM $CuSO_4$ for 8 hours is no longer evident in the presence of TEPA (50 µM) in normoxic condition. The up-regulation of GPER mRNA expression induced by hypoxia (2% O2 for 8 hours) is no longer evident in the presence of TEPA (50 µM) but rescued culturing cells in the aforementioned hypoxic condition along with 200 µM $CuSO_4$, as determined by real-time PCR. Values are normalized to the 18S expression and shown as fold changes of mRNA expression induced by treatments also upon hypoxia respect to cells cultured with vehicle (-) in normoxic condition. (○), (●) indicate $p < 0.05$ (E) In SkBr3 and HepG2 cells, the transactivation of a GPER promoter plasmid

(pGPER) observed upon treatment with 200 µM $CuSO_4$ for 12 hours is no longer evident in the presence of TEPA (50 µM) in normoxic condition. The transactivation of a GPER promoter plasmid (pGPER) induced by hypoxia (2% O_2 for 12 hours) is no longer evident in the presence of TEPA (50 µM) but rescued culturing cells in the aforementioned hypoxic condition along with 200 µM $CuSO_4$. The luciferase activities were normalized to the internal transfection control and values of cells receiving vehicle (-) and cultured upon normoxia were set as 1-fold induction upon which the activities induced by treatments also in hypoxic conditions were calculated. Each data point represents the mean ± SD of three independent experiments performed in triplicate. (○), (●) indicate $p < 0.05$

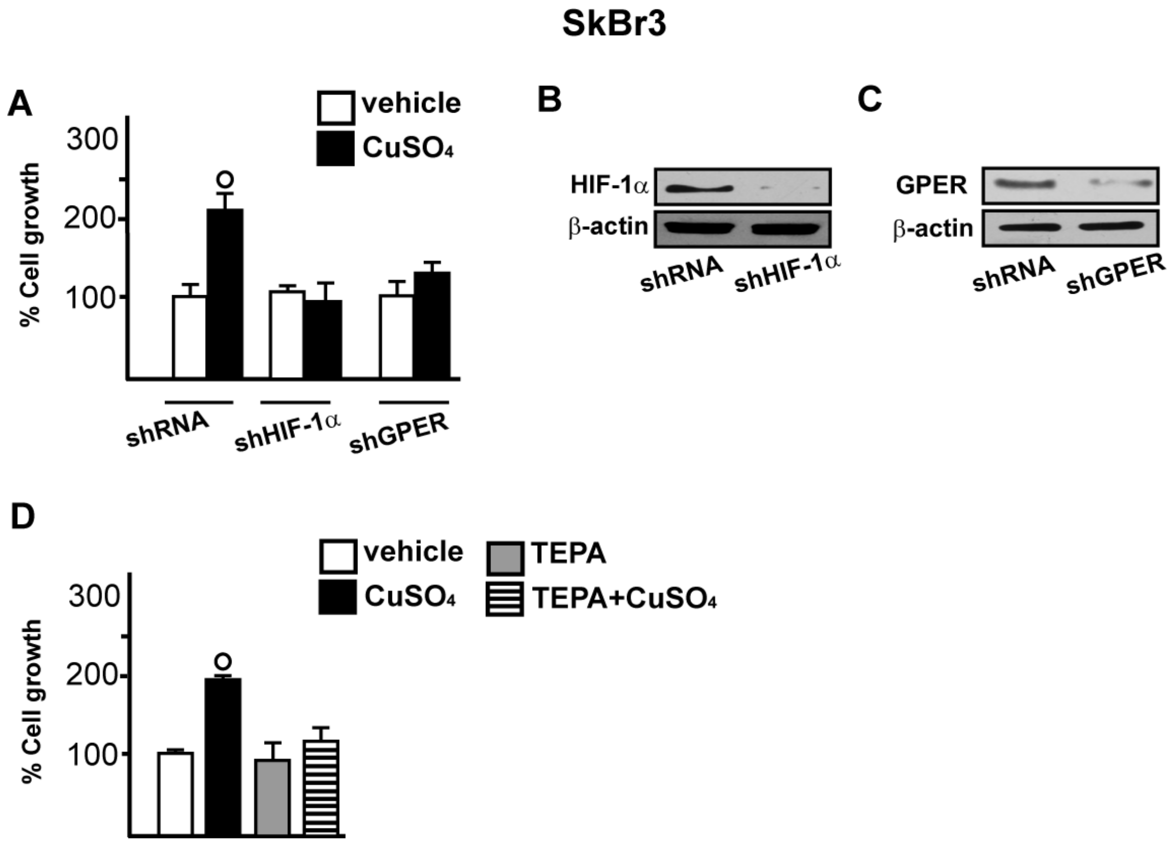

Supplementary Fig. 2: *HIF-1α and GPER are involved in the proliferative effects induced by $CuSO_4$.* (A) MTT proliferation assays in SkBr3 cells transfected for 24 h with shRNA, shHIF-1α or shGPER and then treated for 48 hours with vehicle or 200 µM $CuSO_4$, as indicated. Efficacy of HIF-1α (B) and GPER (C) silencing in SkBr3 cells. (D) MTT proliferation assays in SkBr3 cells treated for 48 hours with vehicle, 200 µM $CuSO_4$ alone or in combination with TEPA (50 µM). Values are mean ± SD of three independent experiments. (○), $p < 0.05$ for cells receiving vehicle versus $CuSO_4$ treatment.
